# Use of Artificial Intelligence and mobile applications in the early detection of suicide risk among young people across the globe

**DOI:** 10.1192/j.eurpsy.2026.10681

**Published:** 2026-07-31

**Authors:** M. C. B. Maia, B. E. Bandeira, L. A. Brito

**Affiliations:** Universidade de Brasilia, Brasilia, Brazil

## Abstract

**Introduction:**

Youth suicide is a major global public health issue, the fourth leading cause of death for ages 15–19, according to graphic 1 (Hughes et al. J Adolesc Health 2023; 72:367–375). Incidence rises from age 10, especially in males (3:1 ratio). Early detection is difficult due to multifactorial causes; depressive symptoms and subtle behavioral changes are often missed. With 85% global wireless access, mobile apps and AI offer continuous monitoring and personalized interventions. This study reviews their contributions and limitations, including mood monitoring, voice analysis, and social media analysis, evaluating usability, effectiveness, and ethical challenges.

**Objectives:**

This study evaluates mobile apps and AI for early suicide risk detection in youth, reviewing tools like mood monitoring, voice analysis, geolocation, and social media. It assesses effectiveness, usability, and ethical challenges, highlighting their potential to prevent suicide and support mental health.

**Methods:**

A literature review (since 2017) used PubMed, SciELO, BMC Psychiatry, SpringerLink, JMIR, and Arxiv. Keywords included ‘suicide prevention’, ‘AI’, ‘mobile health applications’, ‘youth’, and ‘digital phenotyping’. Included studies addressed mobile apps or AI for adolescent/young adult suicide risk; studies outside the population or without digital tools were excluded.

**Results:**

Apps like BeyondNow (Australia), BlueIce (UK), LifeBuoy (Australia), and eRAPPORT (France) show good usability and real-time mood monitoring (Larsen et al. PLoS One 2016; 11:e0152285). Evidence for reducing suicidal ideation exists, but prevention of attempts remains limited (Baldessarini et al. Harv Rev Psychiatry 2020; 28:15–29). As shown on graphic 2 (Witt et al. Lancet Psychiatry 2017; 4:486–500; Torous et al. Lancet Digit Health 2021; 3:e555–e562), AI using passive data (GPS, time at home, voice) and digital phenotyping achieves promising predictive accuracy Latin America (Chile, Brazil, Colombia) adapts global tools or develops local solutions for remote monitoring. According to table 1 (Souza et al. Rev Bras Psiquiatr 2021; 43:609–617; WHO 2022), Europe conducts the majority of robust studies and clinical trials, whereas Latin America prioritises local projects and pilot innovations, grappling with structural challenges such as digital inequality and the necessity of public policies for integration.

**Image 1:**
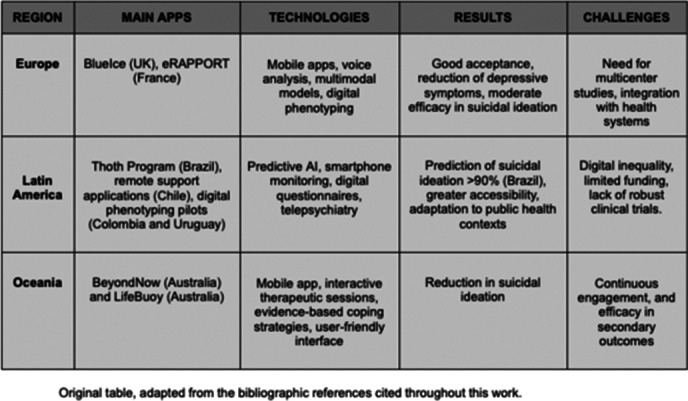
Image 1: Long description.

**Image 2:**
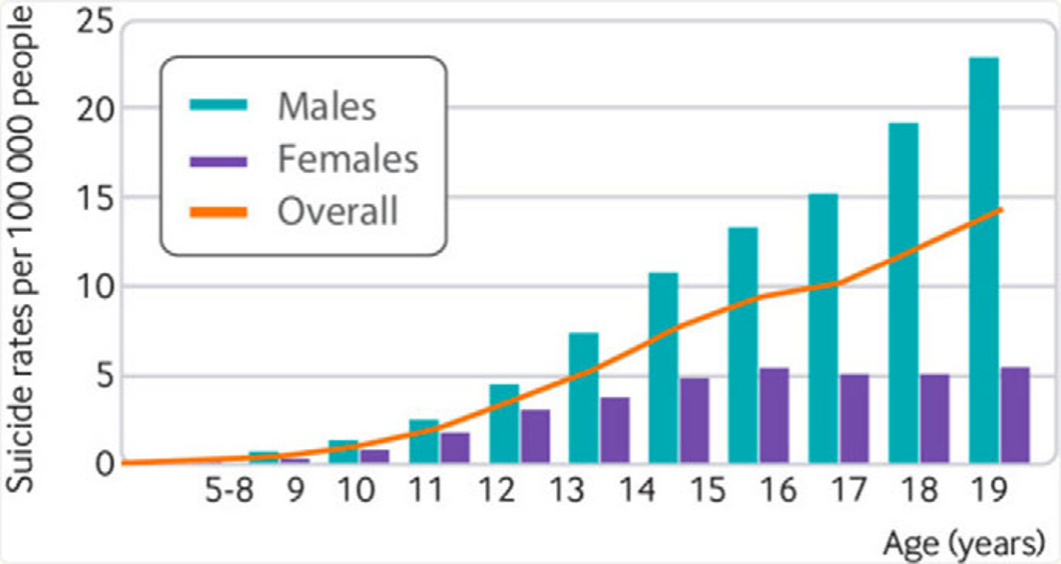
Image 2: Long description.

**Image 3:**
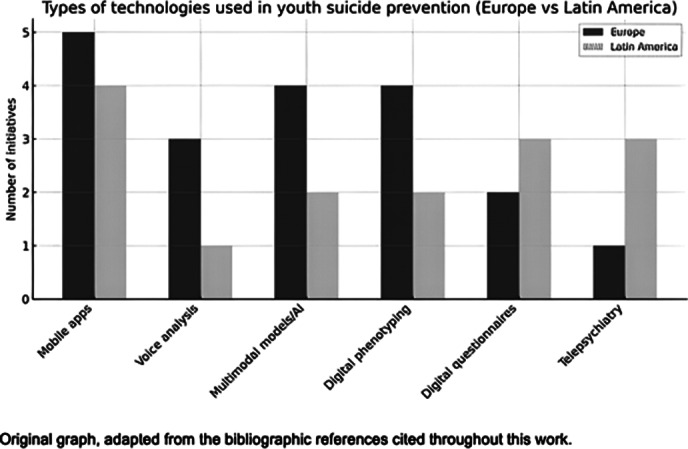
Image 3: Long description.

**Conclusions:**

Mobile apps and AI enable early detection, continuous monitoring, and personalized interventions, but challenges remain: validation, healthcare integration, data protection, and ethics. Hybrid models combining human care and digital tools are key. Europe leads in AI innovation; Latin America focuses on accessible, locally adapted solutions amid digital inequalities and policy needs.

**Disclosure of Interest:**

None Declared

## Long descriptions

### Image 1: Long description

The table consists of five columns and three regional rows.

Row 1: Europe

- Main Apps: BlueIce (U K), eRAPPORT (France).

- Technologies: Mobile apps, voice analysis, multimodal models, digital phenotyping.

- Results: Good acceptance, reduction of depressive symptoms, moderate efficacy in suicidal ideation.

- Challenges: Need for multicenter studies, integration with health systems.

Row 2: Latin America

- Main Apps: Thoth Program (Brazil), remote support applications (Chile), digital phenotyping pilots (Colombia and Uruguay).

- Technologies: Predictive A I, smartphone monitoring, digital questionnaires, telepsychiatry.

- Results: Prediction of suicidal ideation greater than 90 percent (Brazil), greater accessibility, adaptation to public health contexts.

- Challenges: Digital inequality, limited funding, lack of robust clinical trials.

Row 3: Oceania

- Main Apps: BeyondNow (Australia) and LifeBuoy (Australia).

- Technologies: Mobile app, interactive therapeutic sessions, evidence-based coping strategies, user-friendly interface.

- Results: Reduction in suicidal ideation.

- Challenges: Continuous engagement, and efficacy in secondary outcomes.

Navigate back to Image 1:.

### Image 2: Long description

The x-axis represents Age in years, ranging from 5-8 to 19. The y-axis represents Suicide rates per 100 000 people, with increments of 5 from 0 to 25. A legend in the top-left identifies teal bars as Males, purple bars as Females, and an orange line as Overall.

* Males (teal bars): Show a significant exponential increase starting from near 0 at age 5-8 to approximately 23 at age 19. The growth accelerates sharply after age 13.

* Females (purple bars): Show a much slower linear increase, starting near 0 at age 5-8 and reaching approximately 5.5 at age 19. The rate remains relatively flat between ages 15 and 19.

* Overall (orange line): Tracks between the two bar series, showing a steady upward trend from 0 at age 5-8 to approximately 14 at age 19.

Data points for age 19 show the widest gap, with male rates being more than four times higher than female rates.

Navigate back to Image 2:.

### Image 3: Long description

The Y-axis is labeled Number of initiatives and ranges from 0 to 5. The X-axis lists six technology categories. Dark gray bars represent Europe and light gray bars represent Latin America.

* Mobile apps: Europe has 5 initiatives and Latin America has 4.

* Voice analysis: Europe has 3 initiatives and Latin America has 1.

* Multimodal models/A I: Europe has 4 initiatives and Latin America has 2.

* Digital phenotyping: Europe has 4 initiatives and Latin America has 2.

* Digital questionnaires: Europe has 2 initiatives and Latin America has 3.

* Telepsychiatry: Europe has 1 initiative and Latin America has 3.

Europe leads in mobile apps, voice analysis, multimodal models/A I, and digital phenotyping, while Latin America leads in digital questionnaires and telepsychiatry.

Navigate back to Image 3:.

